# Precipitation Inhibition by Small-Molecule Analogues To Sustain Drug Supersaturation

**DOI:** 10.1021/acs.molpharmaceut.6c00245

**Published:** 2026-04-28

**Authors:** Parag Roy, Oisín N. Kavanagh

**Affiliations:** School of Pharmacy, 5994 Newcastle University, Newcastle upon Tyne NE1 7RU, U.K.

**Keywords:** ciprofloxacin, supersaturation, pH-shift dissolution, fluoroquinolone analogues, nucleation inhibition

## Abstract

Maintaining supersaturation during gastric-to-intestinal pH transitions can be essential to maximize the oral exposure of pH-sensitive drugs. This study demonstrates a mechanistically guided strategy to stabilize ciprofloxacin supersaturation using structurally related fluoroquinolone analogues as nucleation inhibitors. Structural similarity screening identified danofloxacin and levofloxacin as promising candidates, and their effects were evaluated using nucleation induction time, dynamic pH-shift dissolution, and ^1^H NMR spectroscopy. Danofloxacin produced the most significant extension of nucleation induction time and was superior to classical polymeric precipitation inhibitors (hypromellose and polyvinylpyrrolidone). During pH-shift dissolution, ciprofloxacin alone retained only ∼20% of its initial concentration after 30 min at pH 7.0, whereas danofloxacin and levofloxacin sustained ∼92% and ∼37%, respectively. Correspondingly, AUC_0–240_ min increased by 5.44-fold for danofloxacin and 1.65-fold for levofloxacin, with HPMC and PVP providing 2.8-fold and 1.25-fold increases. ^1^H NMR revealed concentration-dependent aromatic shielding consistent with ciprofloxacin···analogue heteroassociation, with danofloxacin producing the most substantial and most symmetric perturbations. These data sets support a kinetic mechanism in which isostructural analogues disrupt ciprofloxacin self-association, delay the formation of prenucleation aggregates, and prolong supersaturation. The findings establish small-molecule structural mimicry as a viable pathway for stabilizing supersaturation and guiding mechanism-based coformulation design.

## Graphical abstract

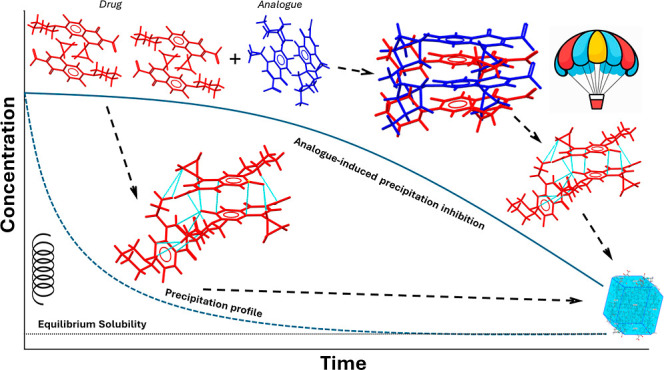

## 1 Introduction

During the administration of weakly basic drugs, the transfer of gastric contents from the stomach to the intestinal tract can induce supersaturation and subsequent precipitation.^1^ We are beginning to understand the importance of this endogenous supersaturation generation, which (if not enabled) can lead to incomplete absorption.^2^ Then, strategies to maintain supersaturation during gastric-to-intestinal pH transitions could maximize the exposure of pH-sensitive drugs.^3–6^ Classical polymeric precipitation inhibitors such as hypromellose (HPMC) and polyvinylpyrrolidone (PVP) are widely used to sustain supersaturation of these drugs by engaging in nonspecific hydrogen bonding/hydrophobic interactions.^7,8^ Although this strategy is effective at relatively low loadings in some systems (≤1% w/w),^9,10^ its performance remains formulation-dependent, often requiring a relatively high quantity of polymeric excipients, which can lead to large, bulky dosage forms and cause physical instability issues due to their hygroscopic nature.^11^ In this study, we evaluate a novel, mechanism-guided approach to sustain supersaturation that targets the earliest solution-phase steps of nucleation, drawing on evidence from our earlier work.^12,13^

Ciprofloxacin (CIP) is a second-generation fluoroquinolone antibiotic generally used to treat urinary, respiratory, and gastrointestinal infections.^14^ CIP exhibits a bioavailability of ∼69% with a typical oral dose of 500 mg, and a minimum aqueous solubility of 0.1989 mM (65.83 mg/L), placing it in BCS Class IV.^15^ As an amphoteric drug, CIP’s solubility and dissolution depend significantly on solution pH. Shifting the pH of a solution with >0.1 M CIP from acidic to neutral pH will decrease the equilibrium solubility (with p*K*_a_ values near ∼6.1 for the carboxylate and ∼8.7 for the piperazinyl nitrogen) and create a transient supersaturated state, followed by rapid nucleation and precipitation. In vivo, ionization in the early gastric environment will temporarily increases the apparent solubility of CIP, but the subsequent rise in pH across the gastrointestinal tract will drive a supersaturation–precipitation transition.^16^ This behavior makes CIP an appropriate model drug for examining supersaturation-stabilization mechanisms. Under gastrointestinal pH-shift conditions, the degree of supersaturation influences the dissolution kinetics, thereby shortening the window for intestinal absorption unless stabilized.^17^ Our approach to maintaining CIP supersaturation is based on isostructural molecules that compete with CIP self-association and delay the formation of critical nuclei.^12^

In our previous work with ocular CIP formulations, we observed that a rapid pH-shift (from pH 4.5 to 7.0, mimicking tear fluid dilution) induces crystallization and precipitation of CIP in the cornea and that this can be inhibited with a set of carefully selected inhibitors.^12^ The present study investigates whether similar principles could be useful to sustain supersaturation during gastrointestinal transit. The structural similarity between CIP and the fluoroquinolone analogues was assessed using the SwissSimilarity platform to construct an analogue database, after which nucleation induction time (*T*_ind_) experiments and dissolution studies were performed.^18^ HPMC and PVP were included as classic precipitation inhibitors which can benchmark our novel strategy against established methods. In addition to the dissolution studies, proton ^1^H NMR spectroscopy was employed to investigate and understand the noncovalent interactions underpinning the anticrystallization phenomena in solution.^19–21^ ^1^H NMR studies allowed direct observation of chemical shift perturbations, providing qualitative and quantitative insights into heteroassociation of CIP with its analogues and self-association across controlled pH and ionic strength.^22^ By combining dissolution behavior with the solution-state analysis, we explored how structural similarity influences the maintenance of supersaturation and validated a mechanism-based coformulation strategy to improve the dissolution performance of pH-sensitive drugs during gastrointestinal transit.

## 2 Materials and Methods

### 2.1 Materials

Ciprofloxacin (free base) and the fluoroquinolone analogues levofloxacin, ofloxacin, fleroxacin (FLE), danofloxacin (DAN, as mesylate), along with hypromellose and polyvinylpyrrolidone (≥98% purity) were purchased from Tokyo Chemical Industry Co. and Fluorochem Ltd. and used without further purification. Deuterium oxide (99.9 atom % D, with 0.05 wt % 3-(trimethylsilyl)­propionic-2,2,3,3-d4 acid, sodium salt), phosphate buffer saline (PBS) tablets and reagents were purchased from Sigma-Aldrich. All solutions were prepared with fresh type II deionized water.

### 2.2 Preparation of Solutions

For CIP control experiments, stock solutions were prepared by dissolving 200 mg of CIP in 250 mL PBS, adjusted to pH 4.5 with 1 M HCl. The solution was stirred overnight to ensure complete dissolution. For coformulation experiments, CIP was combined with fluoroquinolone analogues at a 1:1 molar ratio. Danofloxacin and levofloxacin were used as received, and the pH of each mixture was adjusted to 4.5 to ensure dissolution. Each mixture was stirred overnight to fully dissolve the solids. Formulations with classical inhibitors (HPMC and PVP) were also prepared at a 1:1 weight ratio and prepared under the same conditions.

### 2.3 Nucleation Induction Time

Inhibitor screening experiments were performed in a 1.5 mM (supersaturation ratio, σ = 6.6) CIP solution in saline, with fluoroquinolone analogues at a 1:1 molar ratio, and with HPMC and PVP at a 1:1 weight ratio (Figure 1). To ensure complete dissolution, 1 M HCl was added dropwise to adjust the pH of each solution to 4.5, and the solutions were stirred overnight at room temperature. To induce a pH shift, 5 mL aliquots of each solution were transferred into 10 mL glass vials and stirred at 700 rpm. A calculated volume (8–20 μL) of 0.5 M NaOH was added to each vial to shift the pH to 7, simulating the gastrointestinal pH-shift to record the induction time, which was defined as the moment at which nucleation was visible (*n* = 10).^23^

**Figure 1:**
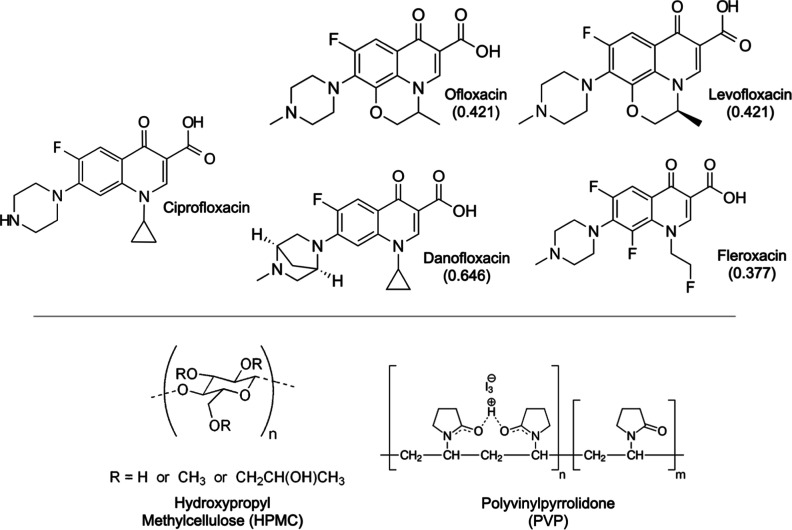
Structure of the selected inhibitors with fingerprinting score inset.

### 2.4 Dynamic pH-Shift Dissolution Studies

Dynamic pH-shift dissolution studies were conducted in a USP Apparatus II (paddle method) to simulate the gastrointestinal transit. All tests were performed at 37 ± 0.5 °C with a paddle rotation speed of 100 rpm. To mimic the elevated gastric pH observed postprandially, the dissolution vessels were charged with 250 mL of the preprepared stock solutions in PBS at pH 4.5, as described in Section 2.2. After 30 min of stabilization, the pH was rapidly increased to ∼7.0 by the addition of 5 M NaOH, simulating the transition to the small intestine during gastric emptying. At predefined time points (0, 10, 20, 30, 40, 50, 60, 75, 90, 105, 120, 180, and 240 min), 1 mL aliquots were withdrawn using a syringe and passed through a 0.45 μm PTFE filter to remove any precipitated solids. Each withdrawn sample was immediately replaced with 1 mL of PBS, pH 7.0, to maintain a constant dissolution volume. Samples were immediately diluted and analyzed to record the concentrations over time.

### 2.5 Quantitative Analysis

Concentrations of CIP and the analogues in solution were determined by high-performance liquid chromatography (HPLC). An Agilent 1260 HPLC system with a UV–vis detector (λ = 280 nm) and a Kromasil C18 reversed-phase column (5 μm, 250 × 4.6 mm) was used throughout the entire analysis. For CIP and analogues, the mobile phase consisted of a mixture of acetonitrile and 0.1% v/v trifluoroacetic acid in water, with a flow rate of 1 mL/min. A gradient program was adjusted to ensure clear resolution between the two component peaks in the chromatogram with retention times of 15.2, 16.2, and 17.3 min for LEV, CIP and DAN, respectively. The injection volume for each sample was 50 μL. Calibration curves for CIP and each analogue were prepared (*R* ^2^ > 0.99) and used to quantify dissolved drug concentrations. All dissolution experiments were performed in triplicate; data are reported as mean concentration ± standard deviation at each time point.

### 2.6 Solid-State Characterization of Precipitates

The solid precipitates formed after 24 h were collected from the dissolution vessels, following completion of the experiment. The contents were vacuum filtered, and the retained solids were dried at room temperature. The dried precipitates were analyzed by powder X-ray diffraction (PXRD). PXRD patterns were recorded on a PANalytical Empyrean diffractometer using Cu Kα radiation (λ = 1.5418 Å), scanning from 5° to 40° 2θ at 0.02° step size. The crystalline phases of the precipitates were identified by comparing PXRD patterns to reference patterns for known CIP solid forms.

### 2.7 Nuclear Magnetic Resonance Spectroscopy

^1^H NMR spectra were recorded on a Bruker Avance III HD 700 MHz spectrometer at 25 °C; chemical shifts are quoted in ppm relative to tetramethylsilane. CIP, DAN, and LEV solutions were prepared in deionized water adjusted to pH 4.5, respectively. Spectra were obtained for CIP alone and in variable molar mixtures with DAN or LEV to examine concentration-dependent changes. The solutions were then diluted in Deuterium oxide (99.9 atom % D, containing 0.05 wt % 3-(trimethylsilyl)­propionic-2,2,3,3-d4 acid, sodium salt). Data were analyzed to assess spectral shifts indicative of intermolecular interactions.

### 2.8 Scanning Electron Microscopy

For SEM analysis, samples were mounted on carbon tape and sputter-coated with a gold–palladium alloy for 90 s at a current of 20 mA. The coated samples were then imaged using a high-resolution field-emission scanning electron microscope (Hitachi SU-70, Hitachi, Japan) operated at an accelerating voltage of 2.5 kV and a working distance of 10 mm.

## 3 Results & Discussion

With the knowledge that precipitation inhibitors (PI) for CIP can be identified by structural similarity,^12^ we employed the Extended-Connectivity Fingerprint, diameter 4 (ECFP4), embedded within the SwissSimilarity platform to define molecular similarity^18^ for a range of CIP analogues which can be easily purchased from chemical suppliers, results of this screen are listed in Table 1. ECFP4 was selected because its local, substituent-level structural resolution captures the functional-group patterns and aromatic features most relevant to CIP self-association and analogue-mediated interactions.

**Table 1:** Molecular Fingerprint Similarity Scores and Nucleation Induction Times for Screened Analogues and Polymeric Precipitation Inhibitors

samples	molecular fingerprint score	*T*_ind_ (minutes, mean ± S.D.)
Ciprofloxacin	1.000	1.1 ± 0.11
Ciprofloxacin: Danofloxacin	0.646	56.7 ± 7.13
Ciprofloxacin: Levofloxacin	0.421	2.8 ± 0.20
Ciprofloxacin: Ofloxacin	0.421	2.9 ± 0.17
Ciprofloxacin: Fleroxacin	0.377	-
Ciprofloxacin: HPMC	-	2.6 ± 0.32
Ciprofloxacin: PVP	-	1.5 ± 0.34

CIP exhibits pH-dependent solubility, and supersaturation can be induced by pH shifts, which can occur in the gastric tract. We identified a suitable supersaturation (σ = 6.6) that can facilitate the evaluation of our proposed set of nucleation inhibitors, with turbidity appearing within ∼1.1 min after a pH shift. Table 1 summarizes the average *T*_ind_ values for CIP alone and in combination with each PI (mean ± SD, *n* = 10). The presence of either a fluoroquinolone analogue or PI substantially delayed the onset of CIP crystallization for all PIs. LEV and OFL also increased *T*_ind_, although to a lesser extent (∼2.8, 2.9 min) than DAN, which substantially extended *T*_ind_ with no turbidity or precipitate observed until ∼56.7 min. Supersaturation induction experiments could not be performed with FLE due to the precipitation of the less soluble fleroxacin hydrochloride monohydrate (CCDC Refcode: XUGKOZ) at pH 4.5 (Supporting Information).^24^ Nucleation inhibition was also observed with polymer PIs, with a *T*_ind_ of ∼1.5 min for PVP and ∼2.6 min for HPMC. The extent of nucleation inhibition followed the order DAN > LEV = OFL ≈ HPMC > PVP > CIP, this sequence aligns with the structural similarity between the CIP and the fluoroquinolone analogues. DAN, which is structurally most similar, produced the longest *T*_ind_, suggesting that specific molecular interactions could interfere with CIP self-assembly. Although their aqueous solubilities vary (Supporting Information), both LEV and OFL delay *T*_ind_ similarly, indicating that the inhibitory effect depends on structural similarity rather than solubility parameters (LEV is the active S-enantiomer of OFL).^25^ On this basis, only LEV was selected as the representative lower-similarity analogue for mechanistic comparison.

Since the environment and conditions can significantly influence observations in supersaturated states, we believed it was important to determine whether this extension to induction time was also observed after pH shifts, which are used in two-stage in vitro dissolution assays typically employed to assess precipitation in the gastric tract.^26,27^ In these studies (Figure 2), we again found that CIP precipitated rapidly upon transitioning to a higher pH, with more than 80% of the initial concentration precipitating within 30 min of the pH shift, rapidly approaching the CIP equilibrium solubility minima (pH 7.0). Co-formulation with structurally similar fluoroquinolone analogues (LEV and DAN) significantly altered the dissolution profile of CIP. The sharp decrease in CIP concentration after the pH shift was prevented by the presence of the analogues. After 30 min postshift, CIP alone retained only ∼20% of its initial concentration, while coformulation with LEV retained ∼37%, and DAN retained a notable ∼92%. Similarly, classic PIs slowed the concentration decline; HPMC retained ∼86% and PVP ∼25% of CIP’s initial concentration after 30 min of pH-shift. These trends highlight the superior performance of DAN and HPMC in sustaining CIP supersaturation. Remarkably in the CIP-DAN coformulation, no visible precipitate appeared even after 4 h, indicating extended metastability of the supersaturated solution. Precipitation was eventually observed at 24 h, indicating that the system remains thermodynamically unstable but kinetically stabilized during the critical absorption window. Analysis of the precipitates obtained from the dissolution studies confirmed the formation of CIP hydrate by PXRD, illustrated by the characteristic long needles of the channel-hydrate family, consistent with previously reported forms such as ENODUH (4.8-hydrate) and YAPMEJ (3.7-hydrate), which are known to vary only subtly when evaluated by PXRD.^28,29^

**Figure 2:**
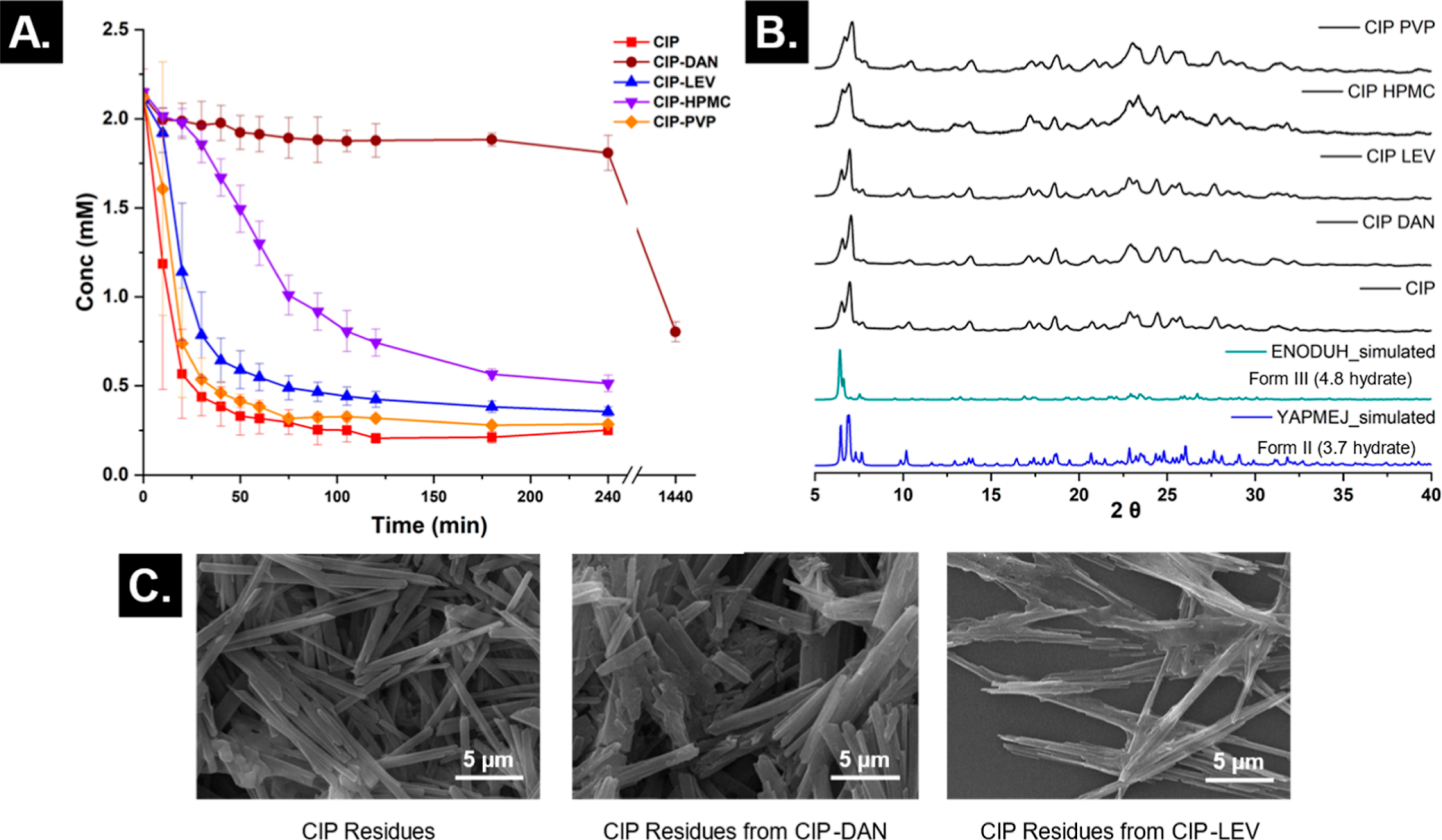
(A). Dynamic pH-shift dissolution behavior of CIP alone and in the presence of inhibitors, (B). Comparative PXRD of the residues with reported forms. (C). SEM micrographs of the residues.

Quantitatively, the area under the concentration–time curve (AUC_0–240_ min) was substantially enhanced for all coformulations: ∼5.44-fold higher for CIP-DAN, ∼1.65-fold for CIP-LEV, ∼2.8-fold for CIP-HPMC, and ∼1.25-fold for CIP-PVP, relative to CIP alone. Interestingly, despite CIP-HPMC’s *T*_ind_ being similar to CIP-LEV, its AUC exceeded that of CIP-LEV. In contrast, the fluoroquinolone analogues improved dissolution parameters without requiring polymers or surfactants, emphasizing a distinct mechanistic pathway for inhibiting nucleation via specific intermolecular interactions, which can be selected for in a design strategy.

To complete our understanding of how these structurally similar fluoroquinolones influenced the solution-state behavior of CIP, we examined concentration-dependent perturbations in the ^1^H NMR resonances of CIP in the presence of increasing concentrations of DAN and LEV (Figure 3). This provided direct insight into how the fluoroquinolone analogues influence the solution-state behavior of CIP. Individual ^1^H NMR spectra of CIP, DAN and LEV with corresponding peak assignments and interpretation are provided in the Supporting Information. CIP displays three well-resolved aromatic protons in the 6–8.5 ppm region, corresponding to the quinoline group. Across the entire concentration series (CIP: analogue = 1:0.1–1:10), all three aromatic resonances shifted progressively upfield, indicating systematic shifts in their local magnetic environments upon association with either DAN or LEV. This is likely to be due to π–π interactions.^30^ In the presence of DAN, the CIP’s aromatic protons exhibited apparent and concentration-dependent upfield shifts. At equimolar concentration, CIP resonances shifted by −0.10, −0.09, and −0.14 ppm, and these perturbations increased to −0.30, −0.22, and −0.40 ppm at 10-fold DAN excess. DAN resonances also shifted, but to a smaller extent (−0.07, −0.11, −0.04 ppm at 1:1; −0.26, −0.32, −0.15 ppm at 1:10). This CIP-centered perturbation pattern shows that DAN could progressively displace CIP–CIP contacts as DAN concentration increases. LEV produced a similar but less pronounced effect on the CIP spectral profile. CIP aromatic protons shifted to a lesser extent (−0.01, −0.04, 0.12 ppm at 1:1; −0.07, −0.09, −0.33 ppm at 1:10), whereas LEV’s own aromatic resonances shifted more prominently (−0.15, −0.20 ppm at 1:1; −0.25, −0.46 ppm at 1:10). This inverted asymmetry, where LEV is more perturbed than CIP indicates a weaker association that perturbs CIP’s self-assembly to a lesser extent. NMR titration data were analyzed using a nonlinear least-squares fit to a 1:1 binding model (*quadratic equation*).^31^

1 $$\Delta \delta =\frac{\Delta \delta_{\max}}{2\left[CIP\right]}\cdot \left(\left[CIP\right]+\left[L\right]+\frac{1}{k_{1:1}}-\sqrt{{\left(\left[CIP\right]+\left[L\right]+\frac{1}{k_{1:1}}\right)}^{2}-4\left[CIP\right]\left[L\right]}\right)$$

**Figure 3:**
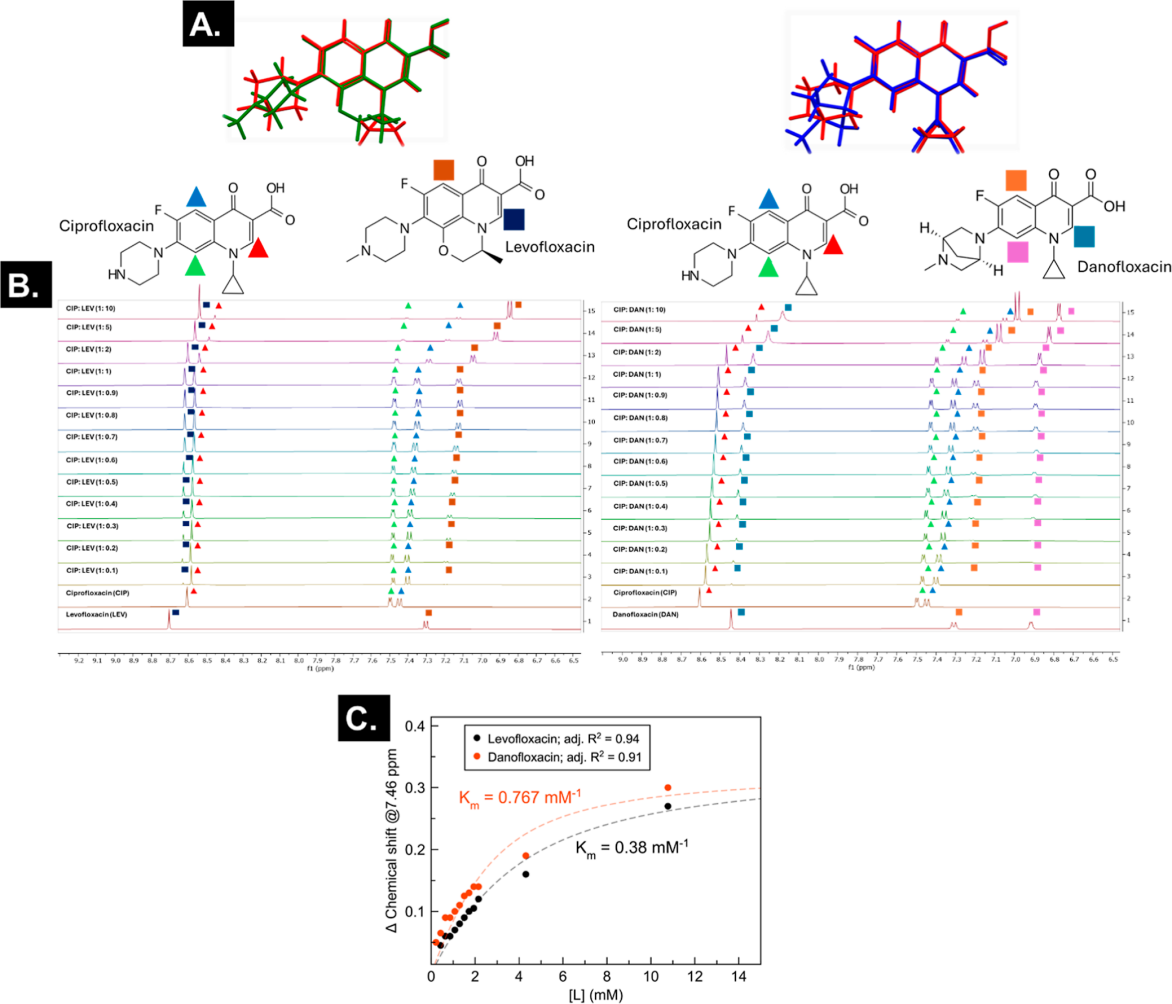
(A). Overlayed structural similarity between CIP (Red), LEV (Green), and DAN (Blue). (B). ^1^H NMR spectra of CIP in the presence of variable molar concentrations of the inhibitors. (C). NMR titration data using a nonlinear least-squares fit to a 1:1 binding model.

The observed chemical shift changes showed good agreement with the 1:1 model (*R* ^2^ > 0.90 for all fitted resonances), which derived K_1:1_ of 0.77 and 0.38 mM^–1^ for DAN and LEV respectively, indicating moderate binding affinity. The progression of aromatic shifts across the concentration range clarifies the functional difference between the two inhibitors. DAN induces measurable CIP shielding even at low ratios and continues to produce increasing perturbations across the series, consistent with the formation of robust CIP-DAN heteroassociates that effectively replace CIP–CIP interactions. In contrast, LEV, although capable of heteroassociation, produces more minor and earlier-plateauing CIP perturbations, suggesting less optimal access to the CIP aromatic region. This spectroscopic behavior aligns directly with the *T*_ind_ and dissolution behavior; DAN’s more substantial aromatic influence correlates with its markedly longer *T*_ind_ and greater supersaturation maintenance, whereas LEV’s lesser impact corresponds to its more modest interaction with CIP.

### 3.1 The ‘Analogue’ Mechanism for Supersaturation Stabilization

Maintaining supersaturation following a ‘*spring’* event is traditionally achieved through PIs.^32^ These polymers may prolong the supersaturated state by increasing solution viscosity raising, the steric and kinetic barriers to nucleation.^33^ Although this strategy can be effective at relatively low loadings in some systems, higher concentrations of PIs can become self-limiting as excessive viscosity may inhibit mass transfer, potentially counteracting the desired inhibition effect. Furthermore, polymer PI efficacy is highly sensitive to the physiological environment, where drug ionization and competition from surfactants or bile salts can destabilize polymer–drug interactions.^34^ In addition, many PIs are highly hygroscopic, absorbing atmospheric moisture that increases molecular mobility and triggers drug recrystallization during storage.^35^ This physical instability is often compounded by phase separation within supersaturating drug delivery systems such as Amorphous Solid Dispersions, where the drug and polymer spontaneously unbind, diminishing the formulation’s solubility advantage.^36^ Practical constraints also include low drug loading capacity, which often forces a high pill burden on patients to maintain the necessary polymer-to-drug ratio for inhibition.^37^ Furthermore, the manufacturing process, whether spray drying or hot-melt extrusion, can introduce thermal or mechanical degradation to sensitive compounds.^34^ While PIs remain highly effective, their limitations create an opportunity for approaches that intervene differently in the nucleation pathway and do not depend on polymer behavior. Structurally related analogues offer such an alternative by modulating the initial molecular interactions that drive self-assembly.

The combined ^1^H NMR, nucleation-induction, and pH-shift dissolution data support a mechanistic model in which structurally related analogues stabilize supersaturated solutions by interfering with the earliest steps of self-assembly in the solution phase. Under pH-shift conditions, protonated CIP molecules tend to self-associate via H-bonding and π-contacts that provide the structural template for the formation of critical nuclei. When a structurally similar analogue is introduced, it competes with CIP for self-association sites, reducing the extent of CIP···CIP interactions that drive early cluster formation. ^1^H NMR aromatic-region perturbations confirm that CIP experiences a progressively altered magnetic environment in the presence of DAN or LEV, consistent with heteroassociation and disruption of native CIP self-assembly. This behavior constitutes an analogue-mediated parachute mechanism, in which the analogue delays nucleation by interfering with CIP’s ability to form the prenucleation aggregates required for crystallization and subsequent precipitation. The result is a prolonged supersaturation period (*parachute* phase), reflected in extended induction times and significantly increased AUC during pH-shift dissolution. DAN, the analogue with the highest structural similarity to CIP, produces the strongest perturbations in solution, resulting in the longest induction times and the most sustained supersaturation. LEV produces similar but weaker effects, demonstrating that the mechanism depends on the analogue’s structural similarity. The ‘*analogue*’ mechanism provides a general molecular approach to stabilizing supersaturation that is mechanistically distinct from classical PIs.

## 4 Conclusions

This work identifies structural similarity as an effective principle for selecting small-molecule inhibitors capable of stabilizing supersaturated solutions during physiologically relevant pH transitions without the need for polymeric excipients. By disrupting the earliest stages of self-association, these analogues delay the formation of nucleation clusters and prolong the supersaturation window that influences dissolution-driven absorption of drug molecules into the systemic circulation. The relationship between structural complementarity, NMR-derived heteroassociation and nucleation induction time highlights a general analogue-based mechanism that complements traditional polymer *parachute* approaches.

Beyond ciprofloxacin itself, these findings expand the formulation toolkit available for challenging oral drug candidates. The ability of one therapeutic analogue to stabilize another sets the stage for multidrug combinations in which drugs of synergistic clinical usage reciprocally sustain other’s supersaturation, offering both pharmacological and biopharmaceutical benefits.^38^ More broadly, we believe that it is possible to combine analogues in a variety of ways. For example, one can envisage that a prodrug, which by nature is structurally similar to the parent, could be a generalizable combination. We also suggest that it is an interesting thought experiment to think about how drugs which are structurally similar (and could have similar pharmacological effects) could be combined in new formulations to adapt physical chemical properties such as supersaturation and solubility. The ‘*analogue*’ mechanism for supersaturation stabilization demonstrated here provides a foundation for structure-guided selection or even rational design of small-molecule nucleation inhibitors for other poorly soluble drugs that suffer from supersaturation-driven precipitation.

## Supplementary Material



## Notes

^1^H NMR spectra, X-ray diffractograms and pH solubility data for the selected fluoroquinolone inhibitors (PDF)
