# Supplementary material for: Precipitation Inhibition by Small-Molecule Analogues To Sustain Drug Supersaturation
Source: Mol Pharm. 2026 Apr 28;23(7):3753–9. doi: 10.1021/acs.molpharmaceut.6c00245 (PMC13343504; doi:10.1021/acs.molpharmaceut.6c00245)
Supplement: Supplementary file 1 [file mp6c00245_si_001.pdf]

Supporting Information

Precipitation Inhibition by Small-Molecule Analogues to Sustain Drug Supersaturation

Parag Roy & Oisín N. Kavanagh*

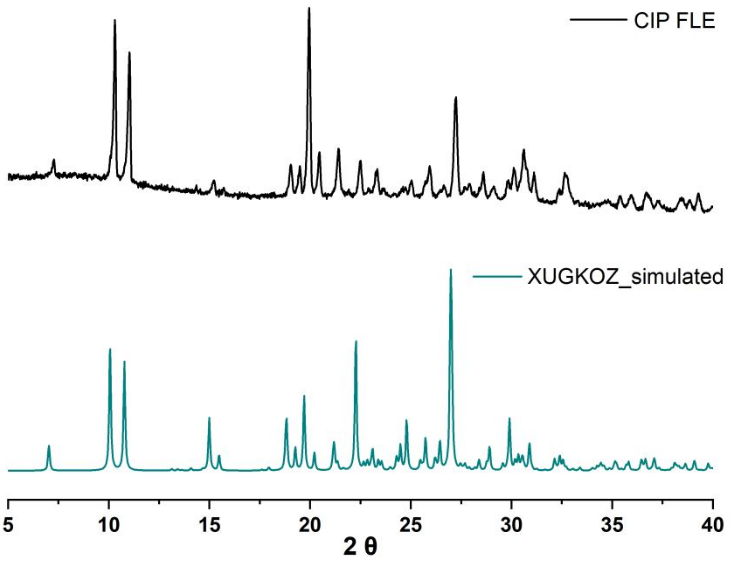

Figure S1: Comparison of the PXRD residues of precipitates from CIP-FLE solution at pH 4.5 and Fleroxacin hydrochloride hydrate (XUGKOZ).

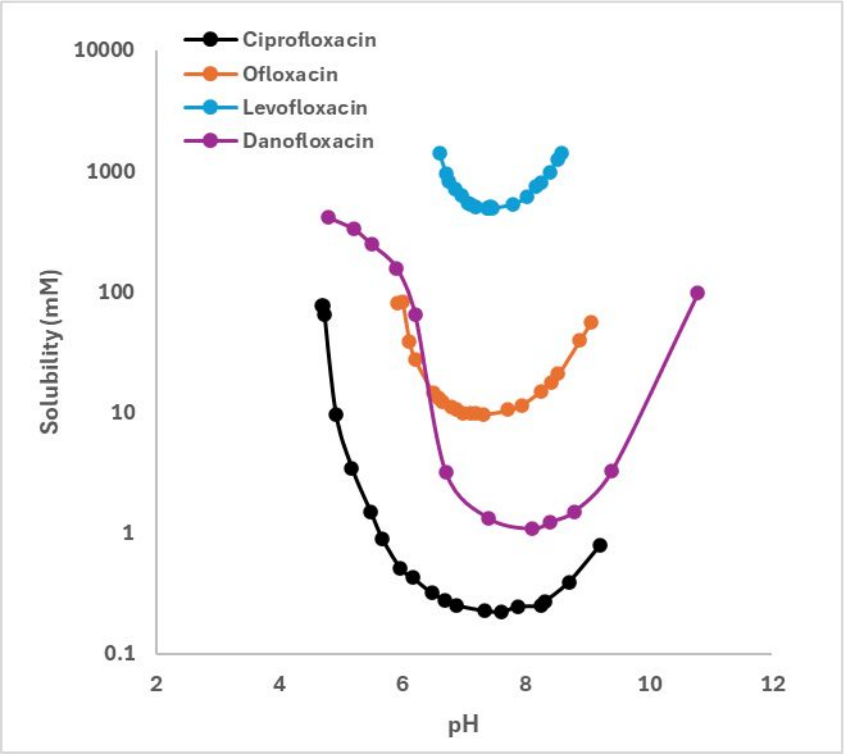

Figure S2: Comparative pH solubility behaviour for Ciprofloxacin, Levofloxacin, Ofloxacin and Danofloxacin.

Ciprofloxacin: ^{1}H NMR (700 MHz, D2O) δ 8.61 (s, 1H), 7.50– 7.49 (d, J = 6.9 Hz, 1H), 7.46 – 7.44 (d, J = 12.9 Hz, 1H), 3.71– 3.70 (t, J = 3.8 Hz, 1H), 3.65– 3.64 (t, J = 5.1 Hz, 4H), 3.55 –3.54 (t, J = 5.1 Hz, 4H), 1.46 –1.45 (d, J = 7.0 Hz, 2H), 1.21–1.20 (d, J = 5.7 Hz, 2H).

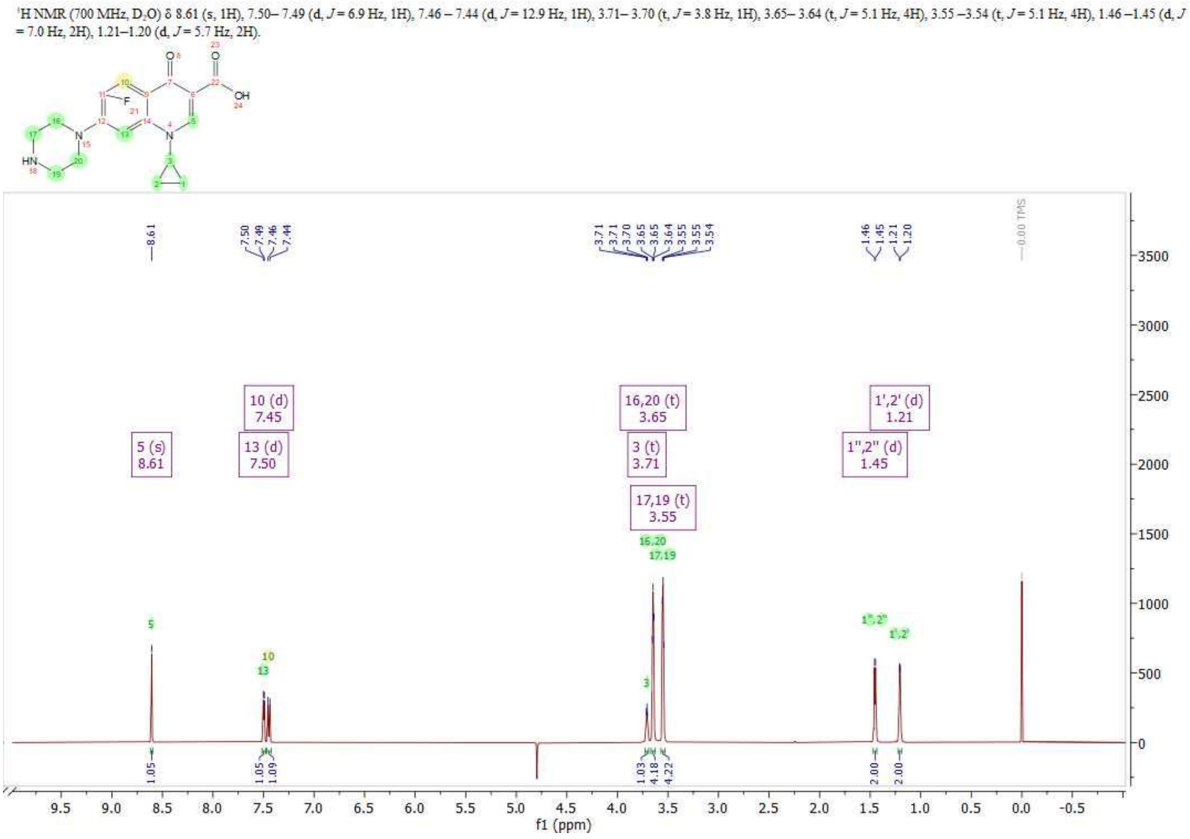

Figure S3: ^{1}H NMR spectra of Ciprofloxacin, with annotations and interpretation.

Danofloxacin Mesylate: ^{1}H NMR (700 MHz, D2O) δ 8.44 (s, 1H), 7.32– 7.30 (d, J = 13.4 Hz, 1H), 6.92 – 6.91 (d, J = 7.2 Hz, 1H), 4.98 (s, 1H), 4.12 (s, 1H), 3.99 – 3.97 (d, J = 11.8 Hz, 1H), 3.71 (s, 1H), 3.60 – 3.57 (m, 1H), 3.37 (s, 1H), 3.08 (s, 3H), 2.82 (m, 4H), 2.48 (d, J = 12.6 Hz, 2H), 1.44 – 1.38 (m, 2H), 1.20 – 1.11 (m, 2H).

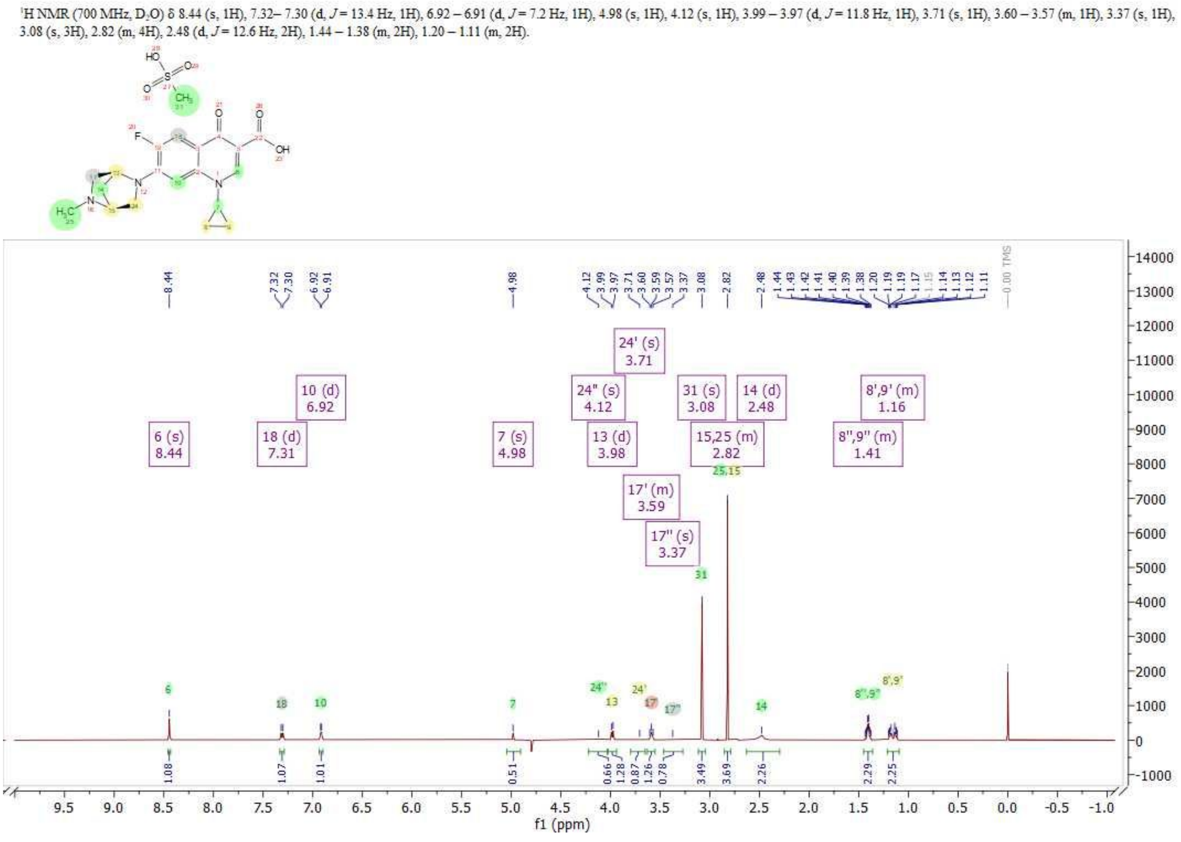

Figure S4: ^{1}H NMR spectra of Danofloxacin Mesylate, with annotations and interpretation.

Levofloxacin: ^{1}H NMR (700 MHz, D2O) δ 8.70 (s, 1H), 7.31 – 7.29 (d, J = 11.9 Hz, 1H), 4.63 – 4.61 (dd, J = 11.7, 2.3 Hz, 1H), 4.50 – 4.47 (dd, J = 11.7, 2.6 Hz, 1H), 3.72 – 3.62 (m, 7H), 3.38 – 3.33 (m, 2H), 3.02 (s, 3H), 1.60 – 1.59 (d, J = 6.8 Hz, 3H).

^{1}H NMR (700 MHz, D_2O) δ 8.70 (s, 1H), 7.31 – 7.29 (d, J = 11.9 Hz, 1H), 4.63 – 4.61 (dd, J = 11.7, 2.3 Hz, 1H), 4.50 – 4.47 (dd, J = 11.7, 2.6 Hz, 1H), 3.72 – 3.62 (m, 7H), 3.38 – 3.33 (m, 2H), 3.02 (s, 3H), 1.60 – 1.59 (d, J = 6.8 Hz, 3H).

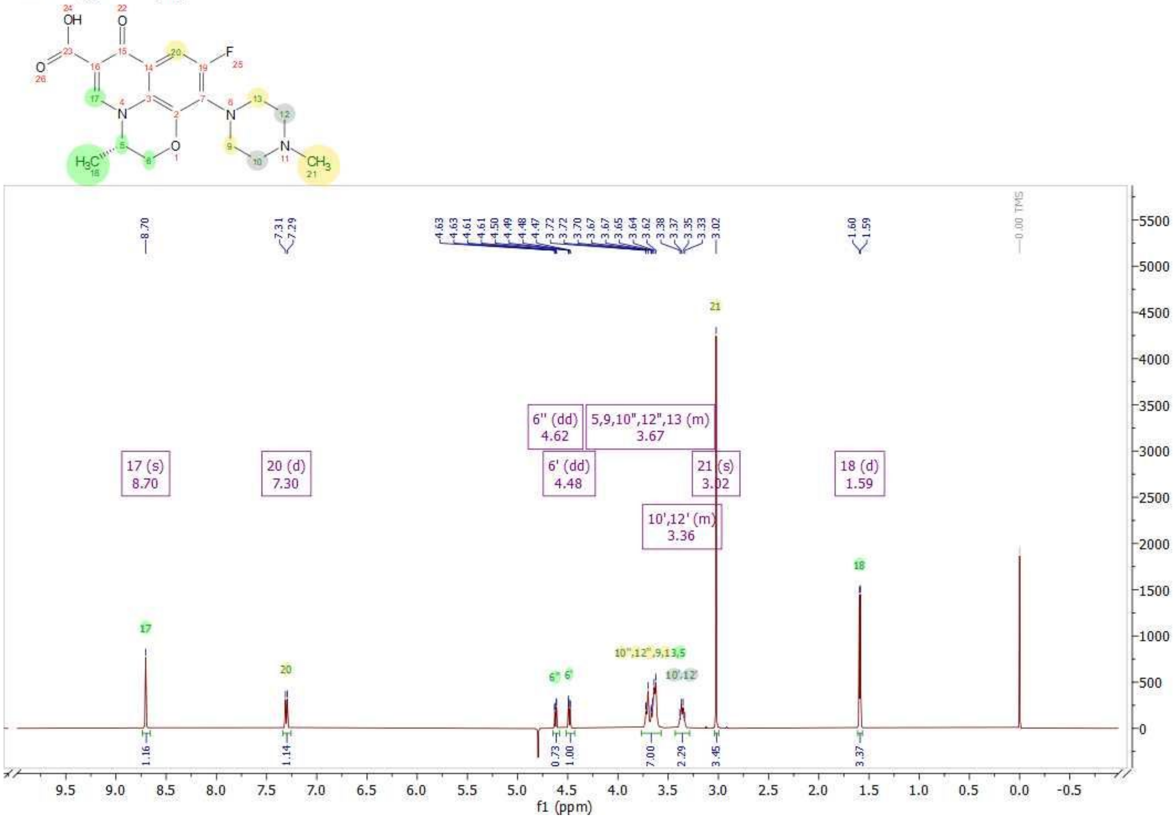

Figure S5: ^{1}H NMR spectra of Levofloxacin, with annotations and interpretation.
